# The Detection of Emerging Trends Using Wikipedia Traffic Data and Context Networks

**DOI:** 10.1371/journal.pone.0141892

**Published:** 2015-12-31

**Authors:** Mirko Kämpf, Eric Tessenow, Dror Y. Kenett, Jan W. Kantelhardt

**Affiliations:** 1 Institut für Physik, Martin-Luther-Universität Halle-Wittenberg, Sachsen-Anhalt, Germany; 2 School of Media and Communication, University of Leeds, Leeds, United Kingdom; 3 Center for Polymer Studies and Department of Physics, Boston University, Boston, United States of America; Fondazione Edmund Mach, Research and Innovation Centre, ITALY

## Abstract

Can online media predict new and emerging trends, since there is a relationship between trends in society and their representation in online systems? While several recent studies have used Google Trends as the leading online information source to answer corresponding research questions, we focus on the online encyclopedia Wikipedia often used for deeper topical reading. Wikipedia grants open access to all traffic data and provides lots of additional (semantic) information in a context network besides single keywords. Specifically, we suggest and study context-normalized and time-dependent measures for a topic’s importance based on page-view time series of Wikipedia articles in different languages and articles related to them by internal links. As an example, we present a study of the recently emerging Big Data market with a focus on the Hadoop ecosystem, and compare the capabilities of Wikipedia versus Google in predicting its popularity and life cycles. To support further applications, we have developed an open web platform to share results of Wikipedia analytics, providing context-rich and language-independent relevance measures for emerging trends.

## 1 Introduction

The majority of people, no matter what their role or position is, exchange a lot of information via peer to peer communication based on messaging services such as Email, Twitter, Facebook, Google+, etc. Background information is often retrieved via web searches and from public web pages, such as the online encyclopedia Wikipedia [1]. *Computational Social Science* as an emerging discipline deals with this new source of social data, trying to merge social sciences, physics and computational sciences to obtain new insights into our social, economic, and political interactions [2, 3]. However, can an increase of interest in a particular topic represented by an online news article or a Wikipedia page be used as an indicator for changes in demand in financial markets, indicate what technology is on the way to be the next rising star, or predict the spreading (epidemiology) of a disease?

Recently, an increasing number of scientific papers use computational social science tools such as Google Trends [4] to identify changes in the web search behavior of the public as indicators of changing public interest in particular topics. After the early success of flu trend predictions since 2008 [5, 6], Google Trends is increasingly used for research in health [7–9], economics [10–14], and other fields [15, 16]. Previous work [17, 18] has suggested that query volume for financial search terms on Google could predict stock market movement. Following this work, Alanyali *et al*. [19] demonstrated a significant correlation between the daily mentions of companies in the Financial Times in the morning and how much they were traded on the stock market during the day. For 2015 alone, we identified about 30 publications which use Google Trends as a main research tool. The results support the hypothesis of an existing influence between public health attitudes or financial markets and the news or social media.

On the other hand, a critical reflection and a quality discussion is more important than just processing more and more data. Lazer *et al*. [20] discuss typical problems and show that mistakes like overfitting a small number of cases, temporal auto-correlations leading to non randomly distributed errors, and a lag of stability of the applied method are critical factors, possibly leading to wrong models and useless predictions. For example, in the case of Google flu trends, the flu prevalence has been overestimated in 100 weeks out of 108 starting in August 2011 [20], and age-related differences in the accuracy of the predictions have been demonstrated very recently [21].

Clearly, many Internet users rely on Google to locate useful information sources online as a starting point and thus generate the data exploited by Google Trends. Some of these searches lead to Wikipedia, which is a widely-used central reference source for background information across a number of subjects. Then, for deeper reading on a specific topic and related subtopics, users often follow links provided by Wikipedia without additional Google searches. Therefore, one can consider Google searches as data that give insights into what information Internet users are initially looking for, whereas Wikipedia access data provide insights into what information Internet users in fact read when they have a deeper interest in a specific topic or field of study. Previous works using Wikipedia for trend evaluation include predicting the spreading of influenza [22] and measuring correlations between stock market data (like trading volume, or volatility) and the access rates to corresponding groups of Wikipedia articles to study the role of protruding social networks in the economic cycle [23] or even to try to use this data to predict future stock market developments [24]. Other studies looked at predicting the box office success of movies [25] and used Wikipedia to study language complexity [26]. The memory effect leading to long-term correlations in hourly Wikipedia access-rate time series, however, seems to be independent of trends [27]. We refer to [28] for a very recent review on trend research using Wikipedia.

In this paper, we have chosen to study the emergence and life cycles of technological trends related to the emerging Hadoop market as an example for demonstrating the potential of Wikipedia in the detection of emerging trends. Our exemplary case study was inspired by life cycle studies, published by Wang in 2006 [29]. Instead of historical sales data we use social media data to measure user interest. Our approach compares data regarding different languages to differentiate between local and global context, which is useful for both behavioral and cultural studies. Specifically, we have initiated and developed *ETOSHA*, an open source software framework for Wikipedia analysis and for studies of collective interests and social trends [30]. Besides the results included in this paper, we have already tested our ETOSHA toolbox in analyzing Wikipedia context networks for Influenza and Ebola, for social network services, and for financial markets [30]. Specifically, we studied data from interconnected financial markets, represented by ten international stock market indexes like Nikkei 225 (Japan), DAX (Germany), NASDAQ100 (U.S.A.), BSE200 (India), and others. Results are visualized as representation plots and relevance plots. Both charts support an advanced interpretation in various interdisciplinary research contexts including econo-physics and socio-physics. The toolbox also provides means to investigate whether changes in the frequency of views of certain Wikipedia pages in English language also anticipate subsequent changes in the interest in the pages about the same topic in other languages. This differentiation defines a local and a global context and allows a comparison of both. Thus, we introduce the first Wikipedia analysis framework, which tracks and captures not what end users are looking for, rather what they are interested in. In the next section, we highlight a few key practical differences between data on Wikipedia usage and data on Google search keyword usage.

### 1.1 Google Trends compared with Wikipedia

Currently, Google Trends seems to be increasingly used in several areas of science, including, in particular, health sciences [6–9, 20, 21], and economic research [10–14, 17–19], to identify changes in attention towards various topics. However, there are several drawbacks of relying on trend time series acquired from Google Trends. Firstly, neither the raw data nor the underlying algorithms are public. For example, the core of Google Flu Trends is a non-public model, which has never been published (to our knowledge) and is currently not visibly maintained [5]. The limitations of the Google Trends’ approaches are also not openly known in general. Changes of the underlying algorithms could be implemented by Google software engineers without public notice, which seems to have occurred in the past [20]. No official Google Trends application programming interface (API) is available to access raw data or Google Trends, hindering a systematic testing and comparison of alternative algorithms. Although several techniques proposed in web blogs and several open source projects enable automated data collection, problems are caused by current undocumented limitations. For example, no more than five keywords can be entered in a standard Google Trends request, although more simultaneous inquiries are possible if a non-official software is used [31].

Secondly, we think that the ambiguity of search keywords can cause misleading results. Many words used as search terms or search keywords are ambiguous; they have multiple meanings. Such limitations often lead to unreliable or unreproducible data sets on which reliable research results can not be built outside the Google space. Since another difference between Google Trends and Wikipedia is the business model behind Google, commercial search term suggestions popping up when keywords are typed in might thus also affect the search process and cause misleading trends. Wikipedia has no such active marketing activities. The Google Trends web application has several limitations, e.g., there is no feature to derive related or semantically correlated keywords in different languages. Furthermore, the keywords as retrieved via the Google-Trends web application provide little insight into which meaning was of interest to the Internet user. Recently Google Trends has begun providing a list of related searches, but a comprehensive semantic context is not provided. In contrast, a Wikipedia page, other than those designed specifically for disambiguation, is about one term only. In some cases, a page redirects to another page with a different name which means the same. Even if a user is not aware of available alternative page names, the interest is counted. Wikipedia redirect pages provide an implicit aggregation of several click trajectories.

Thirdly, according to [4] the Google Trends query index is based on query share, not on an absolute query count. The total query volume for the search term in question within a particular geographic region is thus divided by the total number of queries in that region during the time period being examined. The maximum query share in the time period specified is normalized to be 100 and the query share at the initial date being examined is normalized to be zero. The automated normalization of all results to an interest range from 0 to 100 might hinder easy comparisons of the trend results, since the absolute number of search events a result is based upon cannot be retrieved. The data is available since 2004 at a weekly time resolution. Raw Wikipedia access-rate data, on the other hand, is available since 2007 at hourly time resolution for each individual article (page). The data can be aggregated as needed. On the other hand, Wikipedia access traffic might be considered as a subset of Google search data, since more than three quarters of all users arrive at Wikipedia articles via Google searches [32]. However, we do not claim nor assume that the two systems are statistically independent. An existing dependence is actually positive for our goal of identifying emerging trends from online media. The reason is that because of the dependence all relevant trends—including those emerging in Google—can be captured by analyzing Wikipedia traffic data. An actual drawback for Wikipedia data can be the reduced statistics—traffic on Google is nearly seven times larger than the traffic on Wikipedia [32].

Lazer *et al*. [20] have shown that one has to look clearly on collected data, especially to eliminate bias and to strengthen arguments. In most cases of social media data collection one has no direct control about the raw data collection and as such on the measurement procedure. This makes it even more important to study the systems which provide the data. Precision and accuracy are as important as transparency and stability of the underlying procedures. Independence from unknown external influences is required but difficult to achieve. We suggest that some of the problems with Google Trends could be overcome by focusing on Wikipedia article page view counts instead of Google Trends.

In the following, data from Google Trends [4] and Wikipedia [33] is used as a basis to measure user attraction or user interest in particular topics. Our interest is in the role of Wikipedia as a public crowd-based source for news in the context of market activity, and we are especially focused on the readers side. The number of article downloads reflects the state of a larger part of the society which can hardly be influenced by a dominating opinion of a single publisher using a shiny picture or provocative headlines on page one. Readers select articles intentionally; they are not flooded by topics which just sell well. Our focus on a linguistic and topical context means that keywords or Wikipedia pages are selected according to a given topic. A market study has to cover entities, related to the market, such as participants, competitors, products and also related subjects.

**Fig 1:**
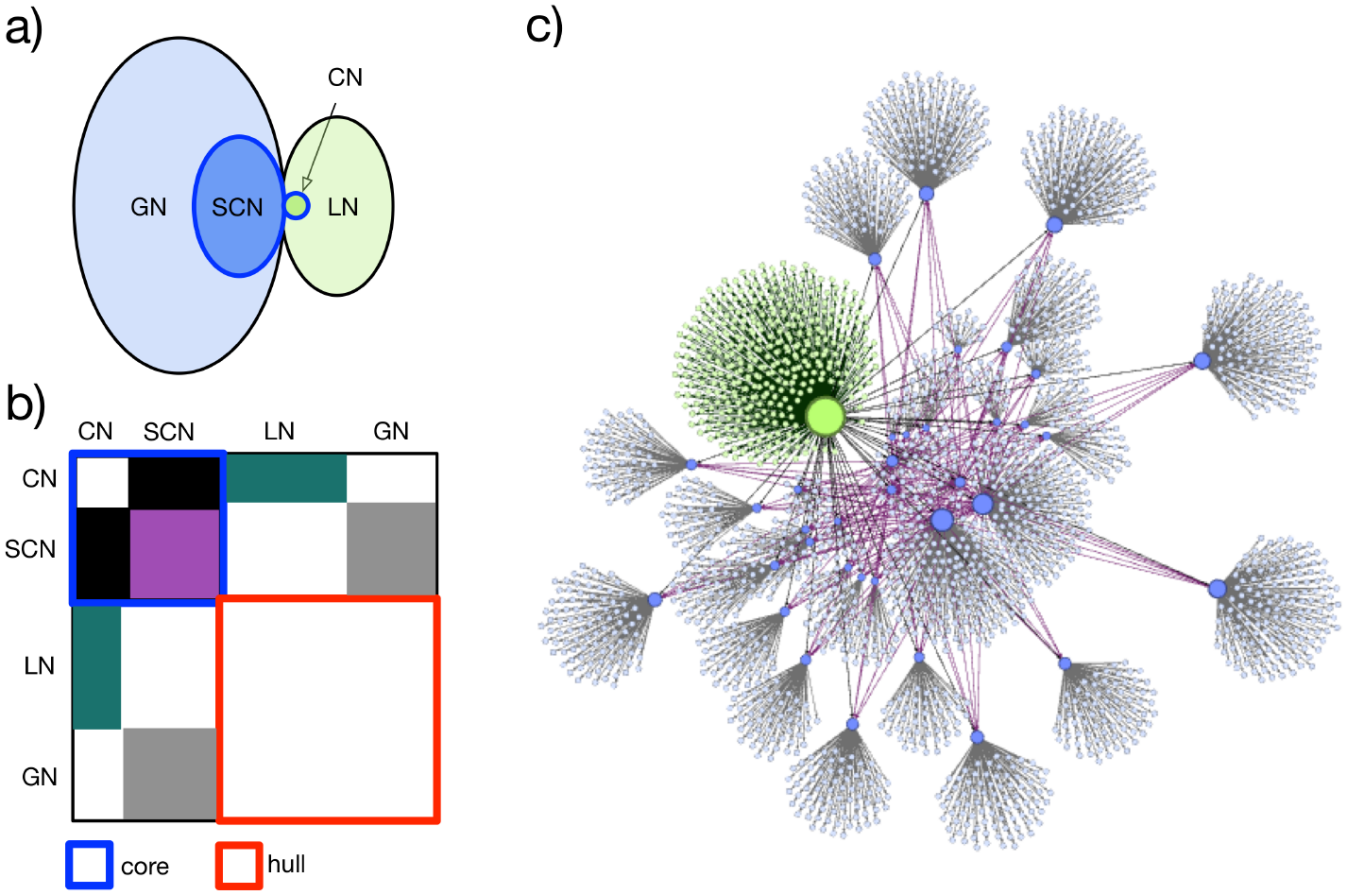
(a) A semantic concept (SC) is represented by Wikipedia pages regarding the same topic in different languages (semantic core nodes, SCN, blue). One node from the SCN, i.e. a specific language, is selected as central node (CN, green) and separated from the SCN. All pages in the same language directly linked to the CN make up the local neighborhood (LN, light green), while all pages directly linked to the other SCN make up the global neighborhood (GN, light blue). (b) Schematic representation of the corresponding adjacency matrix. SCN and CN combined are called multilingual semantic *core*, while LN and GN form the *hull*. The pages in the core are connected via inter-language links (ILL, black for links involving CN and purple for links among SCN). Links between CN and LN are shown in green, while links between SCN and GN are shown in gray. All other links are ignored because only the nearest neighborhood is taken into account. (c) Network representation using the same colors as in parts (a, b). The network representation allows an inspection of the dataset, before group definitions are finalized. A qualitative interpretation and a quantitative measurement of network properties helps to evaluate the impact of separation into sub-networks, e.g. to prepare data for time resolved relevance analysis.

Our approach uses existing implicit semantic relationships between Wikipedia pages to discover such context neighborhoods automatically as illustrated in Fig 1. Based on these local neighborhood networks we have a set of domain-specific topics and the pages which they are embedded in. Using the embedding as kind of a background signal allows us to normalize the directly measured values in a context sensitive and time resolved way. This way, we can identify a broad-based change in interest in a topic that is larger than the change in the interest in one specific page. The approach may provide a middle ground between the very specific study of interest in a particular Wikipedia page and the study of Google Trends in which the search term entered by a user is difficult to disambiguate. Furthermore, using the network surrounding a given topic one should be able to identify more localized differences in the popularity of nodes, if certain aspects of a topic become more or less relevant at certain times.

### 1.2 Wikipedia-based Relevance Indexes

Manually selected Wikipedia pages (see Table 1 in section 2.1) are the entry points for an automatic context aware data collection procedure. These pages are denoted as *central nodes* (CN). Collected data represent the local neighborhood networks (see Fig 1) and contains all immediate neighbor pages (pages directly connected to the page of the CN). The size of the neighborhood is controlled by the link depth parameter *p*_*t*_ with default value *p*_*t*_ = 1. As click count data is only available for the Wikipedia pages and not for all pages in the world wide web (WWW) all external links to such pages are not considered. One term is represented in many languages by individual CNs which form the semantic core (SC). All these CNs for one term can be linked via special links called *inter language links* (ILL) although the ILL graph is usually not fully connected. One particular CN is picked to define a local scope and all other CNs for the same term together we call semantic core nodes (SCN). SCN define the global scope. By doing this we are able to group also the neighborhood pages by a locality attribute. We select the English CN here because of its dominance in IT business. Such an implicit segregation of groups can also be done by topics or other classification approaches using several machine learning algorithms. As such, Wikipedia enables a global and nevertheless language-dependent analysis without the additional effort of parsing, stemming and translating multilingual texts. Additionally, if one is interested in semantic concepts independently of language specific differences, all CNs and their neighborhoods can be aggregated to form a larger data set with better statistics. For details about the structure and creation of the neighborhood networks, see subsection 2.1.

**Table 1:** Selection of English Wikipedia pages (CNs) regarding topics with a direct relation to the emerging Hadoop (Big Data) market. Apache Hadoop is the central software project, beside Apache SOLR, and Apache Lucene (SW, software). Companies which offer Hadoop distributions and Hadoop based solutions are the central companies in the scope of the study (HV, hardware vendors). Other companies started very early with Hadoop related projects as early adopters (EA). Global players (GP) are affected by this emerging market, its opportunities and the new competitors (NC). Some new but highly relevant companies like Talend or LucidWorks have been selected because of their obvious commitment to the open source ideas. Widely adopted technologies with a relation to the selected research topic are represented by the group TEC.

Group	Wikipedia page name (in English Wikipedia project)
**GP**, blue	EMC_Corporation, Microsoft, Oracle_Corporation, IBM, Intel
**HV**, green	Dell, HP, Silicon_Graphics_International*
**EA**, violet	Amazon.com, Facebook, Google, National_Science_Foundation, Yahoo! The_New_York_Times,
**NC**, red	Datameer, BMC_Software, Cloudera, GoPivotal, Hortonworks, Karmasphere, LucidWorks, MapR, Penthao, Sematext, Splunk, Sqrrl, Syncsort, Talend, Teradata, TIBCO, WANdisco
**TEC**, cyan	Java, EMC_Isilon, Sun_Grid, Plattform_Computing, Condor_High-Throughput_Computing_System
**SW**, orange	Apache_SOLR, Apache_Nutch, Apache_ZooKeeper, Apache_UIMA, Apache_Lucene, Apache_Mahout, Apache_Hadoop

**Fig 2:**
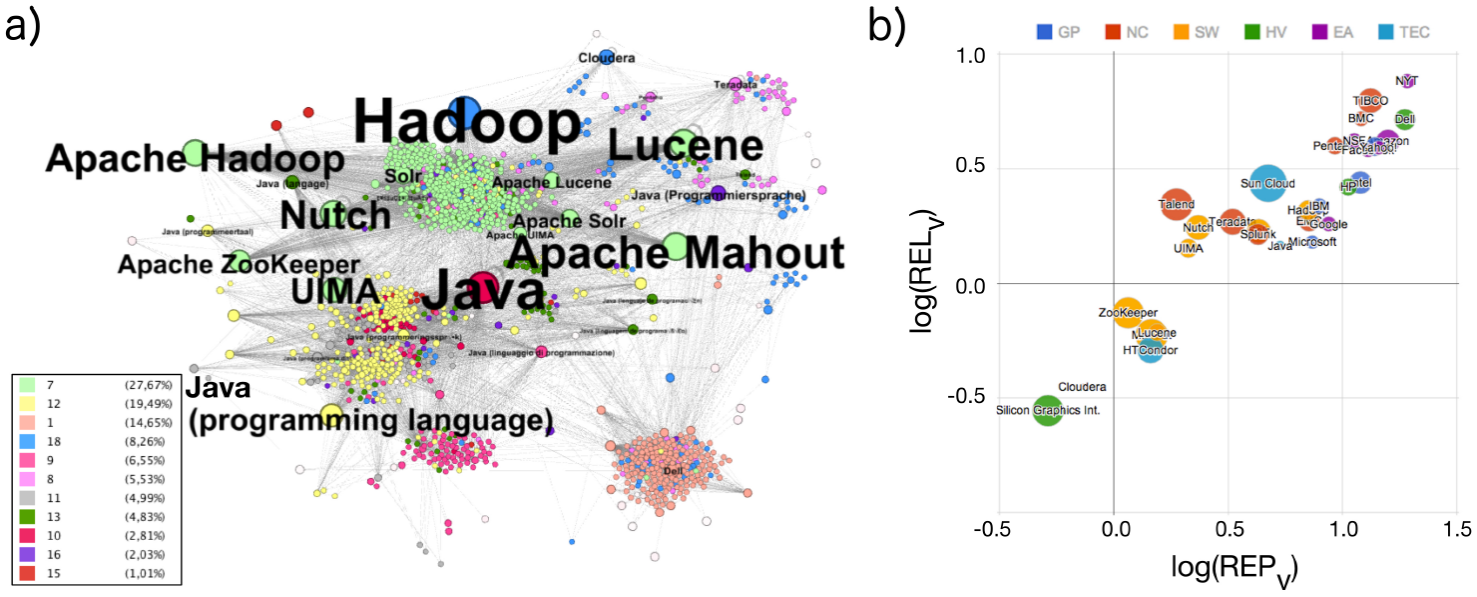
(a) Wikipedia network representation for several interconnected local networks (several CNs with their LNs). The selected topics are related to the emerging Hadoop market, see Table 1 in section 2.1 for details. The colors indicate the membership of the nodes to topical clusters (see legend). Wikipedia pages about Hadoop-related projects are found in close neighborhood of new companies and also close to two important programming languages, Java, and C++, which have both been highly recognized for many years. All CNs with less then five links (*k* = |*LN*|<5) have been removed as the layout was calculated with Gephi (OpenOrd layout). (b) Representation and relevance plot showing text volume REL_*v*_ versus REP_*v*_ with circle sizes given by logREP_*k*_ for all selected CNs as in (a). Colors indicate the role of each group in the emerging Hadoop market and differ from modularity classes used in 2a. One can see a high text volume representation index for pages about the early adopters of Hadoop technology (EA, purple). Wikipedia pages about core technology, which is software such as Apache Lucene, Apache Nutch, and Apache Mahout (SW, orange) have a low relevance index REL_*v*_. Their representation index REP_*v*_ is also small compared with the companies that use the new technology. The label (HV, green) stands for Hadoop-related hardware vendors, (TEC, light blue) for general technology companies, (GP, blue) for global players, and (NC, red) for the competitors within the emerging market.

The analysis procedure consists of the following steps: First, a qualitative analysis of the page content is done to identify the representation bias in the Wikipedia data set. Specifically, we calculate the degree representation index REP_*k*_ of a CN (Wikipedia page) as the ratio of the CN’s degree (number of links to other Wikipedia pages) over the average degree of all SCN pages, i.e., all pages regarding the same topic in other languages. In an analogous way, the volume representation index REP_*v*_ and the user access representation index REP_*a*_ are defined by considering, respectively, the text volumes (numbers of characters) in each page or the total number of downloads of each page instead of the degrees *k*. Hence, the measures REP_*i*_ (*i* = *k* for degrees, *i* = *v* for text volumes, *i* = *a* for user access) compare node-specific properties of the CN with those typical for pages in the considered linguistic and semantic context (the SCN). The details of all numerical procedures are described in section 2.2. Fig 2b shows REP_*v*_ for text volume (horizontal axis) as well as REP_*k*_ for degree (size of circles). A representation bias exists if pages are widely spread in the plot.

The second quantity, used for the vertical axis in Fig 2b is already leading to indications of the considered CNs’ relevances. The degree, volume and access relevance indexes REL_*i*_ (for *i* = *k*, *v*, or *a*, respectively) take into account the local neighborhood (LN) consisting of all pages linked to a CN in the English Wikipedia and the global neighborhood (GN) consisting, analogously, of all pages linked to the SCN in all their languages (see Fig 1). As described in more detail in section 2.2, REL_*i*_ is the ratio of L.REL_*i*_, the representation of the CN compared with the LN, and G.REL_*i*_, the representation of the all SCN compared with the GN.

Next, we calculate the time dependent relevance indexes L.TRRI_*a*_ and G.TRRI_*a*_, taking into account the temporal changes in the users’ download activities. L.TRRI_*a*_ and G.TRRI_*a*_ are relative measures to identify the time evolution of a page’s user attraction in a specific context without disconnecting it from the directly linked neighborhood, i.e., they are time-dependent versions of L.REL_*a*_ and G.REL_*a*_, see section 2.2 for the defining equations. These measures allow an identification of time ranges with significant changes in people’s interest in specific topics or companies. L.TRRI_*a*_ and G.TRRI_*a*_ are auto-adaptive: they emphasize small changes of the considered core with respect to the local and global neighborhoods no matter if the whole system is in an equilibrium or not.

### 1.3 The Emergence of the ‘Big Data’ Market

The emerging high-tech software market we choose as a case study is also called ‘*Big Data*’ market. The open source software projects around ‘*Apache Hadoop*’ known as the ‘*Hadoop ecosystem*’ can be considered to be a seed for the ongoing developments. The first commercial distribution including Apache Hadoop had been released in 2009. The market was not well defined at this time nor was it in equilibrium. We identified some groups of elements which drive or at least influence its evolution. These are (i) the established companies, (ii) the new fast growing technologies, and (iii) the new companies filling the gaps between the big players and sometimes disturbing them.

The representation plot in Fig 2b shows the static (text-volume based) representation indexes REP_*v*_ and relevance indexes REL_*v*_ of all selected Wikipedia CNs, calculated for plain text pages from term statistics, and the representation indexes REP_*k*_ derived from the link structure as explained in the section 2.2. The static (link-based) page network behind Wikipedia is shown in Fig 2a.

Companies like Dell and Facebook have a strong focus on end users; they are well represented in the media as well as in Wikipedia. This may be one of the reasons for their high representation index. Some other much bigger companies are more enterprise-oriented than customer oriented, e.g., Oracle or IBM. The REL_*v*_ values shown in Fig 2b suggest that companies active in the business to customer (B2C) environment might be characterized by relevance indexes above approximately 0.5, while companies with a stronger business to business (B2B) orientation tend to show lower REL_*v*_ values. However, more studies are needed to establish a clear distinction. We note that the history of companies should probably also be taken into account in this respect. For example, Microsoft and Google were established in 1975 and 1998, respectively, while IBM is more than hundred years old and changed business orientation throughout the years.

Wikipedia pages for open source software projects like ‘*Apache ZooKeeper*’, and ‘*Apache Mahout*’ have a positive logarithmic representation index REP_*v*_ but a negative logarithmic relevance index REL_*v*_. This can be understood by looking deeper at these projects. Both are well established, have a large developer community, and are older than five years. They provide very fundamental core technology but have already shifted to the background of many projects. So both are not attracting end users directly, which means they are not generally adopted by a wide user community like ‘*Apache Hadoop*’ and ‘*Apache SOLR*’. The very high level of representation of the ‘*Apache UIMA*’ project can be explained by its closeness to IBM, also shown in the network representation in Fig 2a. IBM pushed and sponsored the project and finally gave it to the open source community. A negative logarithmic representation index indicates an emerging topic which has not received much attraction yet, compared with its local neighborhood. Especially pages with a very weak embedding (a low *k*) show this property. Based on data from 2014 one can see the Wikipedia page about the company Cloudera at a negative logarithmic relevance and representation indexes. This is an indication of a newcomer in the global market. All other Hadoop vendors had no international representation in Wikipedia in 2014. Because of this, the relevance and representation indexes for those pages could not be calculated and compared.

**Fig 3:**
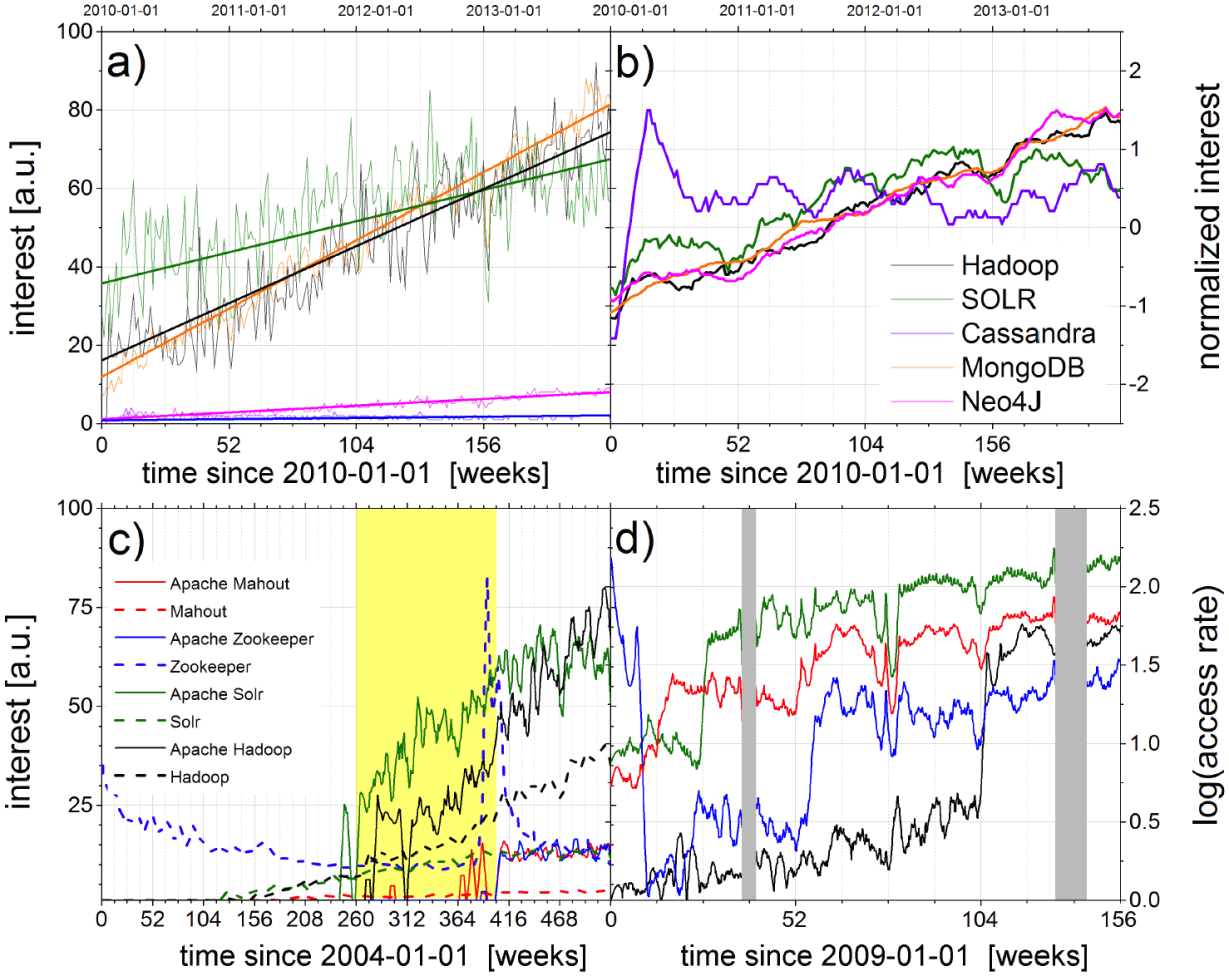
Relative attraction and trends based on Google Trends data (a, b, c) and on access-rate time series from Wikipedia (d) for names of NoSQL databases as search terms (see legends) versus time in months since (a, b) 2010-01-01, (c) 2004-01-01, and (d) 2009-01-01. The approximately linear slopes in (a) differ, and very strong fluctuations are found for Hadoop (black) and SOLR (green), so that the different curves can hardly be compared. In (b) the Google Trends data from (a) have been normalized for zero average and unit standard deviation to facilitate a comparison. However, ambiguous keywords can strongly influence the results as shown in in (c), where raw Google Trends data acquired simultaneously for eight different keywords (using an unofficial API software [31]) is shown. Here, the maximum at interest level 100 (arbitrary units from Google Trends) occurs only for one of the curves in 2015 and is not shown in the plot. Wikipedia access-rate data in (d), corresponding to the time range of the yellow box in (c), indicate a jump in user interest for pages about Apache Hadoop, Apache Zookeeper, and Apache SOLR, which was not visible in the Google Trends data. The gray boxes in (d) indicate times for which no Wikipedia access data is available for technical reasons.

Next, we compare raw and normalized time-dependent data from Google Trends with Wikipdia data to identify trend indicators for market dynamics in the NoSQL data base sector. Fig 3a shows raw data from Google Trends (2010-2014) together with linear fits. The comparison with the corresponding normalized time series in Fig 3b, where the average value is set to zero and standard deviation is set to one, unveils the saturation of interest in the Google Trends data for ‘*Apache Cassandra*’ after an initial peak. The linear trends for ‘*Hadoop*’ and ‘*MongoDB*’ are similar, but differ from the increasing interest trend for ‘*Neo4J*’, if obtained from raw data (see Fig 3a). Normalized data (see Fig 3b) show a comparable trend for all three topics. Unnormalized data underestimate the change in interest for less represented topics. While user interest in the topics *Apache Cassandra* and ‘*Apache SOLR*’ show a saturation and no significant growth during the last years, ‘*MongoDB*’, ‘*Neo4J*’ and ‘*Hadoop*’ constantly attract more interest (with minor differences in seasonal patterns).

Besides normalization, an appropriate selection of domain-specific search keywords has an important influence on the results. According to the goals and research hypotheses of a study one has to avoid insufficient keywords or ambiguous terms. Fig 3c gives an illustration using Google Trends data for the search terms *’Zookeeper’* and *’Apache ZooKeeper’*. Some of the small peaks in the blue dashed curve are caused by events in zoos or movies. For example, the peak in summer 2011 is caused by a movie with no relation to the software project. Wikipedia, on the other hand, uses a special categories to identify pages regarding two or more different topics with the same ‘natural’ page title. This information can be used to define the page selection scope more precisely and also to study its influence on the results of a particular study. Based on this information, Fig 3d shows Wikipedia access rates. It indicates totally different relevances for the terms ‘*Apache Mahout*’ and ‘*Apache SOLR*’ as compared with Google Trends data as shown in Fig 3c. Google trends makes us believe that ‘*Mahout*’ showed up after ‘*Hadoop*’ and ‘*SOLR*’ were popular, but according to Wikipedia the order was different.

## 2 Methods

### 2.1 Defining the Scope: Local Context Networks

In detail, our study of the representation and relevance of the *Hadoop ecosystem* from a multilingual perspective using open social media data begins with the definition of local context networks. Both, Wikipedia content as well as click count data represent user interest in the topic covered by a Wikipedia page. Pages from different topics, like science, art, politics, or pages about commercial entities like companies, brands, or products show different properties, but all are heavily interlinked. Pages differ in size (text volume), node degree (number of links) and also access rate (user download activity) and cannot easily be grouped into disconnected sets. To represent the Hadoop market, we selected a set of 42 Wikipedia pages (CNs) from six categories (see Table 1).

These categories represent multiple facets of the emerging Hadoop (Big-Data) market. There are five global players in IT business (GP, blue) and three hardware vendors (HV, green). Two of the vendors have relevant offerings around Hadoop though one (marked with * in Table 1) has no product in it. Six important early adopters (EA, violet) of the new technology have been selected together with the 17 fastest growing, young start-up companies (NC, red). We added five CNs to cover established and influencing core technologies (TEC, cyan), since the whole field has strong inter-dependencies build on existing business relations. Since most innovations come from open-source software projects (SW, orange) we included seven CNs about Apache. Table 1 shows group names and page names of all selected CNs.

In general, the selection of CNs depends on the topics of a study. In technology market or financial data analyses, for example, one would choose CNs regarding companies, their technology or their products and resources. A good selection of CNs is very important as it defines the scope of the study. In addition, in order to achieve a meaningful interpretation of analysis results all possible influencing factors have to be taken into account—which is impossible in practical applications. Besides this, multiple research disciplines look at different parts of the data set. Therefore, we aim at comparable measurements of nodes’ relevance within local graphs defined by the nodes’ local neighborhoods. Especially local link structures, article lengths, user activity, and editorial activity are considered. The key properties of the method are:

- The local neighborhood defined by explicit links and implicit semantic annotations is examined.
- The context can be defined by a common language or by any other set of semantic concepts. This enables a connection to cultural aspects, related to regions and languages used by specific groups of people.
- The semantic relation between pages in different languages can be used to aggregate data related with a certain topic, e.g. studies related to news, market data, (Twitter) messages, or communication in the context of large important events or movements in societies.

Hence, beside the CNs, their local and global contexts (see Fig 1) have been analyzed. The local context is defined by all articles (nodes) directly linked to the CN in the same Wikipedia, i.e. the same language version. This local neighborhood group of articles is denoted by LN. Each inter-language-link connects the CN to a node addressing the same topic in another language (group SCN). The SCN group defines a global representation of the chosen semantic concept in all languages. We note that inter-language links are not necessarily bi-directional, so that a CN regarding the same topic in another language may have a slightly different SCN group. Our SCN groups are always defined by the inter-language links of the CN. Finally, all articles directly linked to articles in the SCN group form the global neighborhood (GN). This scheme, illustrated in Fig 1, allows aggregation of data regarding specific topics in any language. At the same time, it is possible to separate the whole content stored for a single topic (or term) for each individual language to enable a language-dependent analysis. It is not important if some of the selected pages are also linked to each other within a group or across the group boundaries. The static link structure of the Wikipedia networks was retrieved with the WikiExplorer software [34]. WikiExplorer uses the Java implementation of the Media-Wiki-API published via Google groups under a GPL v3 license.

Besides the link structures of the local and global neighborhoods we have also collected, for each of the articles in groups CN, SCN, LN, and GN: (i) the lengths of the corresponding articles’ texts, (ii) hourly access-count times series, and (iii) edit-event time series. The text lengths were retrieved from Wikipedia as raw wiki-text. The text volume *v* represents the raw numbers of characters of the pages in the original Wiki syntax before the conversion into the more expressive markup language HTML (with much more overhead). The access-count time series data have been extracted from pre-processed data provided by the Wikimedia Foundation [33] using the Hadoop.TS [35] tools. They consist of the number of (user) accesses during each hour between 2009-01-01 and 2011-12-31 (1,095 × 24 = 26,280 data points for each article). We resampled these hourly time series to a daily resolution. Google keyword usage statistics from Google, Inc. have been accessed via the Google Trends web application and via an unofficial Java Google Trends API software [31].

### 2.2 Measuring Context Sensitive Relevance

#### Background and Related Work

Since the numbers of hypertext pages and hyperlinks in the WWW have been continuously growing for more than 20 years, the problem of finding relevant content has become increasingly important. This led, for example, to the growth of Google Inc. with its mission statement ‘to organize the world’s information and make it universally accessible and useful’. Initially, the WWW was mainly a content network and did not reflect relations between authors. It provided structured and connected information. However, the appearance of Social Media Applications (SMA), such as Facebook, LinkedIn, and Twitter, with friendship and follower relations between individual users has led to the creation and simultaneous evolution of novel user community networks (social networks) together with content networks. Such SMAs can be regarded as networks of networks [36, 37], since the underlying user and content networks are closely inter-related with each other. The collaborative creation of linked content became very popular. Another impressive example for this is Wikipedia, a multilingual, web-based, free-content encyclopedia project supported by the Wikimedia Foundation. Because of the intertwined networks involved in the creation and presentation of information in the WWW, the identification of relevant content has become increasingly difficult. Additional problems arise if the time evolution of content relevance shall be traced and if local and global relevance of content shall be distinguished.

Typically, ranking algorithms like the HITS algorithm [38] or (Google) PageRank [39, 40] are used to calculate the relevance or importance of a given page (node) in the WWW. However, ‘relevance’ is not an exactly defined term and cannot be measured in a unique procedure. According to Hjorland *et al*. [41] relevance can be assigned to a thing or to information named **A** in the context of a given task **T**. Only if **A** increases the probability of achieving the goal **G** of task **T**, **A** is relevant to **T**. Without a task and a related goal, relevance does not exist. The identification of relevant information thus requires a context. Here, we use the term ‘relevance’ in the same way as ‘importance’ or ‘meaningful within a given context’. Measuring relevance of a node can be done according to (i) its intrinsic properties (e.g. text length of a WWW page), or (ii) the relative value of an intrinsic property (e.g., text length divided by the average text length of a group of related pages), or (iii) based on structural properties of one of the networks embedding the considered node. PageRank expresses the probability to find a random surfer in a given node [39, 42] and thus exploits mainly the structural properties of the network. Similarly, the HITS algorithm [38] classifies a node either as a hub node (many outgoing links) or as an authority node (many incoming links). Both algorithms are applied to directed graphs and require a dataset describing the full graph. This is very challenging for large systems with billions of nodes.

#### Definition of Representation Indexes

Here, we define and evaluate several parameters that measure the ratio of local representation with respect to global representation for the considered topics. Our first approach is based on the numbers of articles (nodes) in each group, *n*_*LN*_, *n*_*SCN*_, and *n*_*GN*_, see Table 1. Specifically, we define the *local representation index* for node degree by

$$\begin{array}{c} {\text{REP}}_{k}=\frac{n_{\text{LN}}+n_{\text{SCN}}}{r_{\text{GN}}+n_{\text{SCN}}}=\frac{k_{\text{CN}}}{\left\langle k_{\text{SCN}}\right\rangle }\;\text{with}\;r_{\text{GN}}=\frac{n_{\text{GN}}}{n_{\text{SCN}}}. \end{array}$$ (1)

Note that the nominator is identical with the so-called degree *k*_*CN*_ of the CN, while the denominator is the average degree 〈*k*_*SCN*_〉 of all SCN, i. e., the average degree of the node regarding the considered topic in all other languages.

In our second approach, we consider text lengths *v* instead of node degrees *k*. The total text volume per page and the average text volume per group are used as indicators of how well a certain topic is represented within a certain language. We assume that a topic is better represented in the language, in which it has a more comprehensive explanation. Specifically, for the total text volume *v*_*CN*_ of each CN and the total text volume *v*_*SCN*_ of all *n*_*SCN*_ other language versions, we define

$$\begin{array}{c} {\text{REP}}_{v}=v_{\text{CN}}\frac{n_{\text{SCN}}}{v_{\text{SCN}}}, \end{array}$$ (2)

since *v*_*SCN*_/*n*_*SCN*_ is the average text volume of all SCN pages.

Thirdly, we study *time-dependent local representation indexes* based on the time series of the hourly rates of user accesses *a*_*i*_(*t*) or editorial events *e*_*i*_(*t*) for each CN and each node in the corresponding SCN groups. A high number of page views (or editorial changes) can indicate an increased interest of the user community. Although page view data are anonymous, it is possible to use the relationship between a user and his/her preferred language to measure user interest per language. Specifically, for a time slice of width Δ*t* beginning at *t* = *t*_0_, we define

$$\begin{array}{c} {\text{REP}}_{a}\left(t_{0}\right)=\frac{n_{\text{SCN}}\sum_{t=t_{0}}^{t_{0}+\Delta t-1}a_{\text{CN}}\left(t\right)}{\sum_{i\in \text{SCN}}\sum_{t=t_{0}}^{t_{0}+\Delta t-1}a_{\text{SCN}i}\left(t\right)}, \end{array}$$ (3)

where *i* runs over the SCN group corresponding to the considered CN. An analogous definition is used for the editorial events to define *REP*_*e*_(*t*_0_). Clearly, these indexes will be large if there is more user-access activity or more editorial activity, respectively, regarding the CN compared with the averages in other languages.

#### Definition of Relevance Indexes

The local representation indexes (REP_*i*_) are related to a given (or selected) semantic concept, expressed by a Wikipedia page (the CN) in a chosen language and all SCN pages. They indicate how well a topic is *represented* in a given language, irrespective of its embedding within contexts in this language. Only the core of the neighborhood network is considered (see Fig 1a and 1b)). However, it turns out that text lengths, user-access activities, and editorial activities are hardly comparable across different Wikipedias, i.e. across the language versions and cultures. Therefore, more meaningful results can be obtained if we divide by average quantities determined for articles within *the same* language community. Such indexes will characterize how *relevant* a topic is within the selected language or the global context.

Specifically, we study the ratio of the parameters ${L.\text{REL}}_{v}^{\text{LN}}=v_{\text{CN}}n_{\text{LN}}/v_{\text{LN}}$, representing the relevance of the CN in the chosen language, and ${G.\text{REL}}_{v}^{\text{GN}}=v_{\text{SCN}}r_{\text{GN}}/v_{\text{GN}}$, representing the average relevance of all SCN pages within their combined contexts, i.e. the relevance of the selected topic within the other languages. The corresponding *relevance index* is defined as

$$\begin{array}{c} {\text{REL}}_{v}=\frac{{L.\text{REL}}_{v}^{\text{LN}}}{{G.\text{REL}}_{v}^{\text{GN}}}=\frac{v_{\text{CN}}v_{\text{GN}}n_{\text{LN}}}{v_{\text{LN}}r_{\text{GN}}v_{\text{SCN}}}=\frac{\frac{v_{\text{CN}}}{1}\frac{v_{\text{GN}}}{n_{\text{GN}}}}{\frac{v_{\text{SCN}}}{n_{\text{SCN}}}\frac{v_{\text{LN}}}{n_{\text{LN}}}}={\text{REP}}_{v}\frac{\frac{v_{\text{GN}}}{n_{\text{GN}}}}{\frac{v_{\text{LN}}}{n_{\text{LN}}}}. \end{array}$$ (4)

In addition, we compare local *time-dependent relevance indexes* L.TRRI(*t*) with corresponding global time-dependent relevance indexes G.TRRI(*t*) for user-access activity (*a*(*t*)) and for editorial activity (*e*(*t*)) respectively:

$$\begin{array}{c} {L.\text{TRRI}}_{a}\left(t_{0}\right)=\frac{n_{\text{LN}}\sum_{t=t_{0}}^{t_{0}+\Delta t-1}a_{\text{CN}}\left(t\right)}{\sum_{i\in \text{LN}}\sum_{t=t_{0}}^{t_{0}+\Delta t-1}a_{\text{LN}i}\left(t\right)}\;\text{and} \end{array}$$ (5)

$$\begin{array}{c} {G.\text{TRRI}}_{a}\left(t_{0}\right)=\frac{n_{\text{GN}}\sum_{i\in \text{SCN}}\sum_{t=t_{0}}^{t_{0}+\Delta t-1}a_{\text{SCN}i}\left(t\right)}{n_{\text{SCN}}\sum_{i\in \text{GN}}\sum_{t=t_{0}}^{t_{0}+\Delta t-1}a_{\text{GN}i}\left(t\right)}. \end{array}$$ (6)

The width Δ*t* of the considered time slices must be optimized so that random fluctuations are damped while the temporal changes of the relevance indexes remain visible.

## 3 Results: Wikipedia-based Analytics of Project Life Cycles

As an example of possible applications, our analysis has focused on the emerging Hadoop market, starting with an inspection of Google Trends data and single page click-count data from Wikipedia in Fig 3c and 3d. In order to remove biases coming from pages in the close neighborhood or circadian activity patterns [43, 44] we developed the time resolved relevance index.

**Fig 4:**
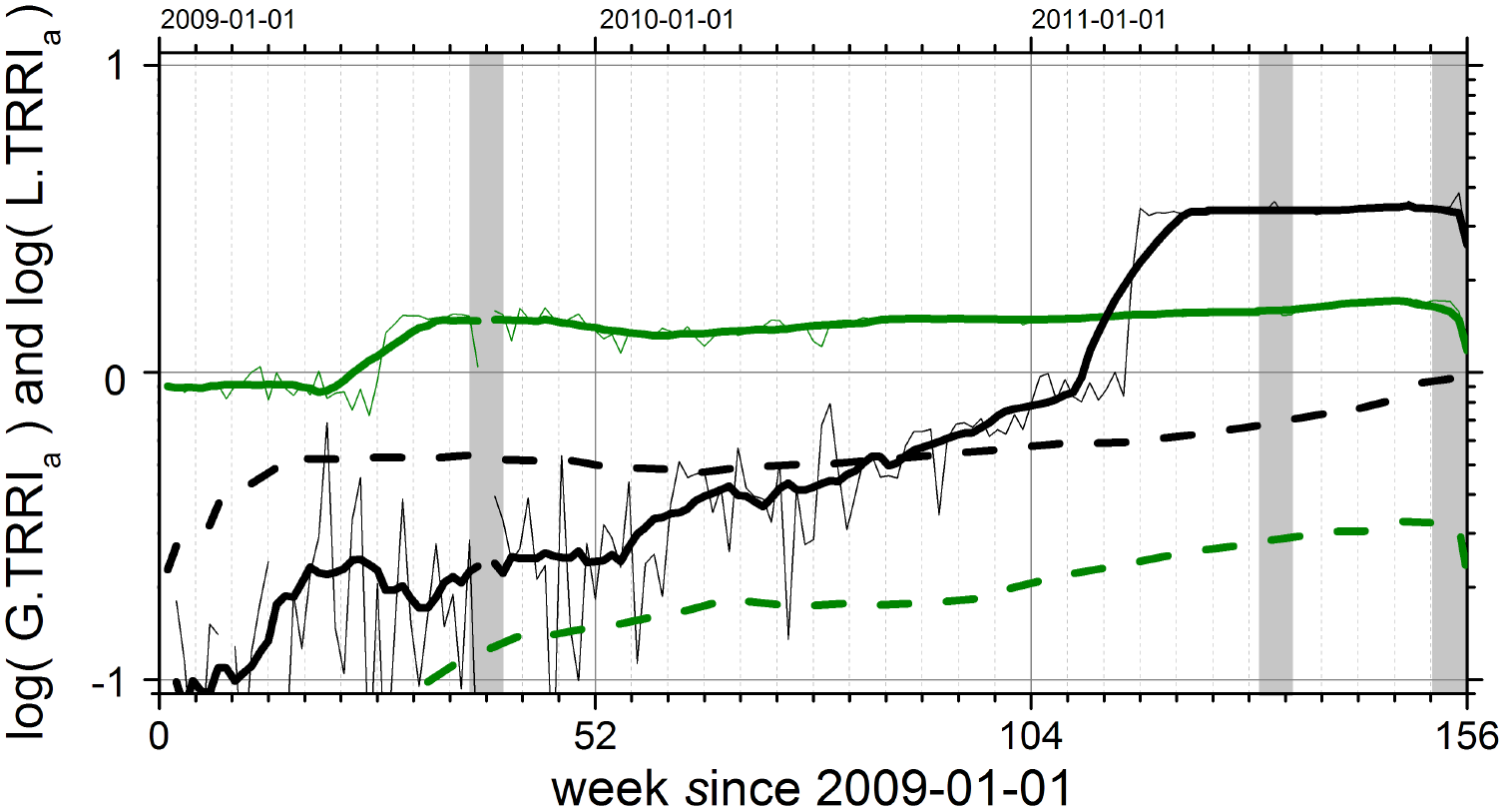
Project life cycle phases derived from Wikipedia usage data based on L.TRRI_*a*_(*t*) (straight lines, Eq (5)) and G.TRRI_*a*_(*t*) (dashed lines, Eq (6)) for Apache Hadoop (black) and Apache SOLR (green) versus time in weeks since 2009-01-01. For Hadoop L.TRRI_*a*_(*t*) we find a strong linear increasing trend with some short-term fluctuations fading out after three months in the years 2009 and 2010. These short-term peaks reflect conference seasons. In 2011, finally, L.TRRI_*a*_(*t*) gets greater than one: a sporadic jump in the user interest is followed by a saturation. G.TRRI_*a*_(*t*) shows a significantly weaker trend during the same time and remains below one. This means that public interest in Hadoop related information is bound to the English language. The ‘break through’ of Apache Hadoop as a relevant topic is thus in 2011, about 18 months after Apache SOLR became a relevant topic. However, finally Apache SOLR is less relevant than Apache Hadoop. The gray areas indicate times for which no data was available for technical reasons. While the thin lines are the original L.TRRI_*a*_(*t*) data, the thick lines have been obtained by applying a running average filter with a window length of 12 weeks.

Fig 4 shows the local (thick lines) compared to the global (dashed lines) TRRI values calculated for daily access rates to Wikipedia pages in the years 2009 to 2011. We consider L.TRRI and G.TRRI values larger than one as highly relevant. In 2009 and 2010, a larger L.TRRI was measured for ‘*Apache SOLR*’, but at the end of our observation period ‘*Apache Hadoop*’ has a larger relevance. Although English is a global language and stands for the global representation of the topic, the label L.TRRI for the English version is used in order to be consistent with Eq (5). Local representation means here: ‘within the IT business context’, while global representation covers all other languages with less importance in the IT sector. The G.TRRI values in Fig 4 (dashed lines) represent the corresponding non-English pages in Wikipedia.

The remarkable change in L.TRRI in the middle of 2009 for the Wikipedia page ‘*Apache SOLR*’ is surprising. The L.TRRI value increased by a factor of two. Earlier, there was a fluctuation around smaller values and after the transition the L.TRRI reached a rather constant level. For ‘*Apache Hadoop*’, an increasing trend can be recognized until L.TRRI reaches one during 2009 and 2010. A jump, followed by a saturation occurs for this page in the first quarter of 2011. In order to identify a recurring generic pattern it is important to analyze more data for the same pages and the following years. Is this saturation level changing again or is there another sporadic increase? Furthermore, other topics, e.g., other technology markets and cultural concepts have to be selected and analyzed this way to figure out if domain or topic specific patterns can be found in the TRRI values. In addition to this analysis procedure we developed and tested another approach, the analysis of functional correlation networks. For this purpose we analyzed the pairwise correlation properties for user activity time series within the neighborhoods before and after jumps. Network layers, which consist of functional links have been calculated. Resulting link strength distributions have been analyzed separately for local and global neighborhoods. We have found a significant change of correlation properties around the jumps. So far we cannot interpret the change in the TRRI value as a cause for the different correlation properties. This results will be the starting point for future research about the dependency between correlation properties and project life cycle phases.

**Fig 5:**
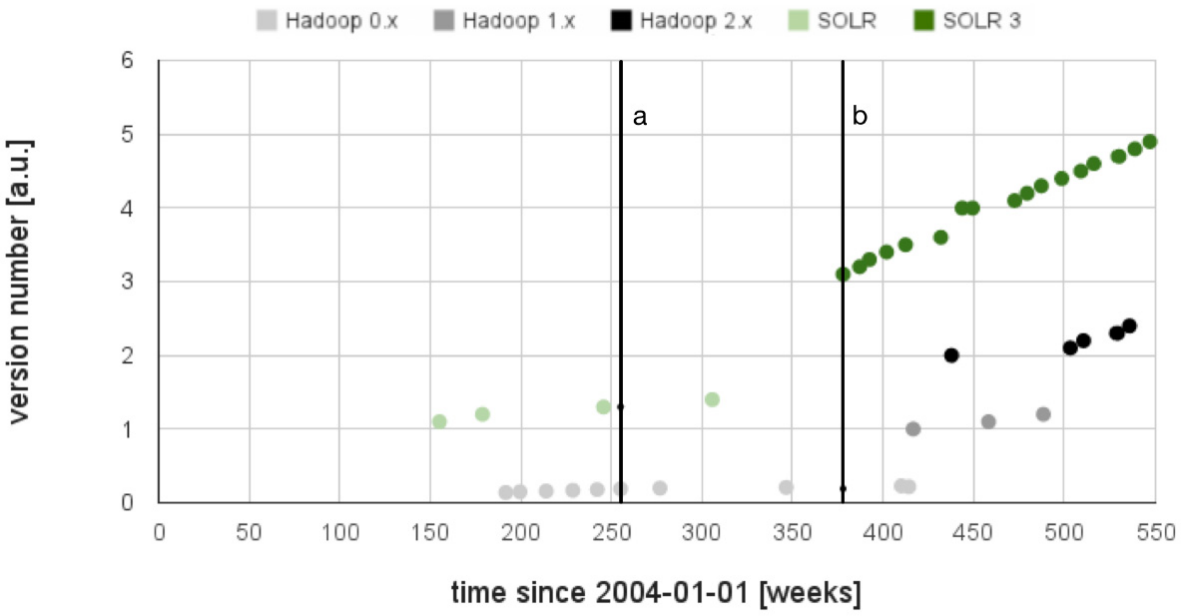
Version number versus release date (in weeks since 2004-01-01) for Apache Hadoop (gray) and Apache SOLR (green). The version numbers for different parallel development branches of both projects indicate ongoing improvements up to the ends of the active projects. The dates of the jump in interest derived from the plot of L.TRRI_*a*_(*t*) in Fig 4 are shown here as vertical black lines for SOLR (a) and Hadoop (b).

One characteristic property of a software project is its release cycle. Fig 5 shows three different coexisting development branches for Apache Hadoop. The two vertical dashed lines indicate the times of the jumps of interest as derived from L.TRRI_*a*_(*t*) in Fig 4. One could expect a positive impact of improvements on relevance or a negative impact of bad software quality. It seems that the new branch in SOLR and Hadoop did not trigger the increase of L.TRRI_*a*_(*t*) values. However, it looks like the SOLR branch starting with version 3 causes an increase of the L.TRRI_*a*_(*t*) for Hadoop. In our special case, the version history allows a contextualization of the results from TRRI analysis: as soon as a critical mass of users exists, TRRI increases, already in early versions. This is a hypothesis for future research on other software projects or new technologies with the goal to quantify this critical mass and the conditions which really lead to a significant increase of L.TRRI_*a*_(*t*) values.

**Fig 6:**
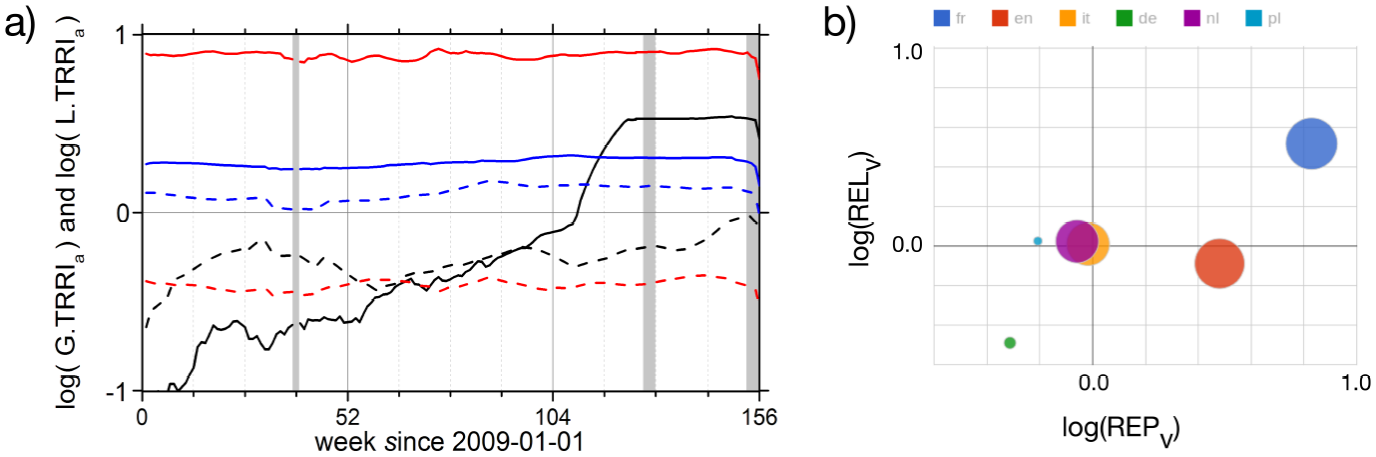
(a) Contextual local and global time resolved relevance indexes L.TRRI_*a*_(*t*) (straight lines, Eq (5)) and G.TRRI_*a*_(*t*) (dashed lines, Eq (6)) for Wikipedia pages regarding the companies Oracle (red), Capgemini (blue) and for Apache Hadoop (black) between 2009-01-01 and 2011-12-31 at weekly resolution. In (b), the text volume representation and relevance indexes are shown (as in Fig 2b) for Capgemini regarding six different languages: the the highest representation is in French. In this case, the local context can be defined as a hybrid context by two CNs—French because of the country of origin, and English because of international IT business. If only one language is used, as in (a), one cannot clearly differentiate between local and global relevance.

Finally, we want to present an example for a language-dependent behavior of relevance indexes. Fig 6a shows a comparison of the global and local time resolved relevance indexes for three CNs. For the companies Oracle and Capgemini we find a stable value while a significant increase in March 2011 is visible for Apache Hadoop. The local relevance starts to dominate over the global relevance after it exceeds the threshold log L.TRRI > 1.0. After this point in time the Hadoop project attracts more user traffic than the pages in its neighborhood. Fig 6b shows an example for strong language dependency. The relevance index for the English page regarding the French company Capgemini is relatively low, while for the other languages, especially for French (but not German), a high REL_*v*_ is measured. One can conclude that an appropriate language version must be selected; otherwise an unknown bias remains. The best context selection is given if the difference between local and global relevance indexes is maximized. Representation plots (as in Fig 6b) can be used as a tool to validate results from TRRI plots (as in Fig 6a), especially in the context of multilingual content networks.

## 4 Discussion and Conclusion

What are the benefits of using the time resolved relevance index TRRI? First, as presented above, in Google search data there exists a hidden bias as it is unknown how other keywords with a strong relation influence trends. In order to get more reliable results, the raw data should be normalized with respect to the local (or global) neighborhood. Such context neighborhoods can easily be derived from Wikipedia page links and multiple Wikipedia projects in different languages. It is possible to uncover all users globally interested in a topic no matter what language they use, because the presented approach automatically takes the semantic neighborhood of a chosen term into account.

However, working with different language version (i.e., different Wikipedia projects) introduces an additional difficulty. Small Wikipedia projects still grow, so the system is not in an equilibrium yet. Large Wikipedia projects, on the other hand, have often reached a saturation, so that different user interest cannot be measured from page properties any more. One could try to analyze editorial activities, but this is also not accurate, as much fewer people are active editors compared to active readers of Wikipedia. According to the published metrics [45], to date, the editor over reader ratio is 1/169 which means that less than 0.6 percent of all readers are active contributors. Using the access-rate data set thus gives us better statistics by two or three orders of magnitude.

Nevertheless, since we suggest relative and time-dependent measures, different group sizes and especially dynamical changes with time can be treated appropriately. Therefore, the results can be compared and related, even though they were obtained for page groups of different sizes, for topics representing different markets, and for different languages. Using such context sensitive relative measures enables interdisciplinary and inter-cultural studies.

Secondly, we can discuss the broad meaning of the obtained results. Companies, such as Yahoo!, Facebook, and Google are not direct competitors in the Hadoop market, because the first two only use (but not develop) the public open-source software Hadoop, while the latter uses a comparable closed-source technology. However, some questions remain. Who will become a global player and who might attract the most Big Data experts? Such questions are important not only for big investors but also for people who want to enter such a high tech market, e.g., by running their own services, or by looking for a valuable position. Using TRRI plots and relevance plots one can identify emerging industrial sectors. More studies like this one have to be conducted for other markets, e.g., automotive, mobile communications, or pharmaceutical companies, or the energy supply sector to obtain more general results.

We have illustrated how Wikipedia data allows tracking public attention and public recognition of emerging topics based on content, contributed by a public crowd consisting of self-motivated editors in a self-organized process, and by access rates which can be considered as a reliable public data source. The introduced approach allows to calculate the time delays between the increases of relevance in the local and global contexts. There seems to be a critical value of L.TRRI, which might be useful as an indicator for a transition in the projects life cycle, e.g., a breakthrough in public acceptance. Thus, it is possible to draw a clear picture of a very young fast growing market. The relevance plot and also the time dependent measures are in line with statements from domain experts and public recognition derived from several information channels, which, however, unfortunately cannot be used for a quantitative comparison. Furthermore, this approach can easily be generalized and extended to analyze content relevance and public recognition in arbitrary types of social communication and content networks. One of the most important technical requirements is availability of content together with editorial history beside access-rate data of at least daily resolution. If those different types of data sets would be available on all web servers the proposed approach could also be extended to any type of web resources. Web servers would have to provide content, collect metadata, and publish such metadata in a reliable way using a consistent API. Especially in the context of the growing linked data cloud it seems to be very useful and promising to combine metadata sets. This allows an investigation of many more interlinked social processes in an increasingly inter-connected society.

### Contextual Information for Open Science

Social media can also be considered as relevant sources of information for research. Following the Open Source, Open Access, and nowadays also the Open Science movement, one can see how important a combined or integrated access to open data is for future research. Data driven studies can be the starting point to integrate several interdisciplinary research methods.

One impressive example was given by Hanke *et al*. recently [46]. They have provided public access to open data sets using established data formats such as [47]. The amount of available public data grows steadily and thus grow the challenges faced by researchers. Beside the research context also data and process management becomes more complex and requires cloud or cluster environments.

Schroeder and Taylor [28] analyzed research activities focused on Wikipedia recently. They have found, that big data research about Wikipedia is related to many different disciplines but remains highly fragmented. One reason can be the absence of a standardized way to access the variety of available data from and about Wikipedia. Our method defines a set of metrics which can be applied to trend studies of any topic if it is represented in Wikipedia. The representation index allows to identify existing biases and the TRRI allows activity based time resolved and context sensitive measure. If obtained in such a way, results are comparable across research domain and languages. Furthermore, it is possible to relate our context sensitive metrics to existing measures form social media analysis, such as the *salience index*, introduced by Segev [48].

Modern IT infrastructures allow processing of very large data sets. Setting up one’s own storage and processing environment locally is a common approach. But in the future, single workstations with traditional analysis tools will not be sufficient any more. Also knowledge management will become a critical task beside mastering the technical skills. Data analysis in remote systems like in the Amazon or Google cloud will become a more common pattern. In both cases, data sets are moved around. This will be inefficient for large data sets, such as the Wikipedia corpus or the full Wikipedia access logs [33].

An alternative option is a hybrid approach. It consists of shared resources and cluster federation. Different analysis clusters are used by multiple research groups with interest in the same data. Only intermediate data sets and results have to be exchanged between those clusters. Technically it will be nearly impossible to copy really large data sets to one’s local system. Even if it has the capacity to handle the data volume, the time to transfer the data will be too long and the cost too high. In many cases it is much more efficient to send an analysis task—as a generic request or even a functional unit (such as an executable portion of software)—to the system which stores the data set. Hadoop clusters use this paradigm successfully, and they thus change fundamental patterns in IT practice. Organizing large open data sets in shared environments could have a major impact on research, especially on interdisciplinary research.

Open access and standard formats are obviously not enough to enable Open Science. Research hypotheses, analysis tools, raw data sets, and result data sets should be connected, not just by citations. We started to host and share our data sets using the Semantic MediaWiki introduced by [49]. As soon as more data sets are inter-linked within a global linked data graph—which for itself is one of the outcome of the ETOSHA project [30]—one can start exploratory data analysis across multiple disciplines, as demanded by [28]. This enables new types of studies and also verification and comparison of results, obtained by a common method from the same data source but in different contexts.

We compared Google Trends and Wikipedia data manually. In the future it will be important to filter key information and to find available data sources for automatic plausibility tests. One contribution towards this goal is the creation of a navigable public data and metadata catalog—a second aspect of the ETOSHA project.

Finally, ETOSHA will be a soft- and hardware independent platform for data, knowledge, and software sharing. We develop data contextualization tools. This means that the representation of topics, e. g. in Wikipedia, is measured and published publicly. If context data is not already available, we store the request and extract the requested context data on demand. Researcher can simply request the context data via the ETOSHA service if installed in their analysis cluster and do not have to run their own data extraction procedures. The metadata catalog creates links between research papers and data sets using data set profiles. Data set profiles allow a comparison and exploration of unknown data sets based on existing studies and their results contextualized by the data set profiles.
